# Supplementary material for: Phenothiazines Inhibit SARS-CoV-2 Entry through Targeting Spike Protein
Source: Viruses. 2023 Jul 31;15(8):1666. doi: 10.3390/v15081666 (PMC10458444; doi:10.3390/v15081666)
Supplement: Supplementary file 1 [file viruses-15-01666-s001.zip › viruses-2450584-supplementary.pdf]

Supplementary Methods

SARS-CoV-2 S-Mediated Cell–Cell Fusion Assay

The inhibitory activities of phenothiazine candidates on SARS-CoV-2-mediated cell–cell fusion were evaluated as previously described [33]. Briefly, HEK293T cells were transfected with pEGFP-N1-SARS-CoV-2-S or pEGFP-N1 as the effector cells by using Lipofectamine™ 2000 Transfection Reagent (Invitrogen). HEK293T, expressing human ACE2 receptors on the membrane surface, were seeded in 96-well plates at a density of 1 × 10^4 for 5 h, followed by adding 293T/pEGFP-N1 or 293T/pEGFP-N1-SARS-CoV-2-S cells with or without compounds. The mixture was co-incubated at 37 °C for 12 h and stained with Hoechst (Thermo Fisher Scientific). Three fields of each compound were randomly selected and observed under the fluorescence microscope.

Supplementary Figures

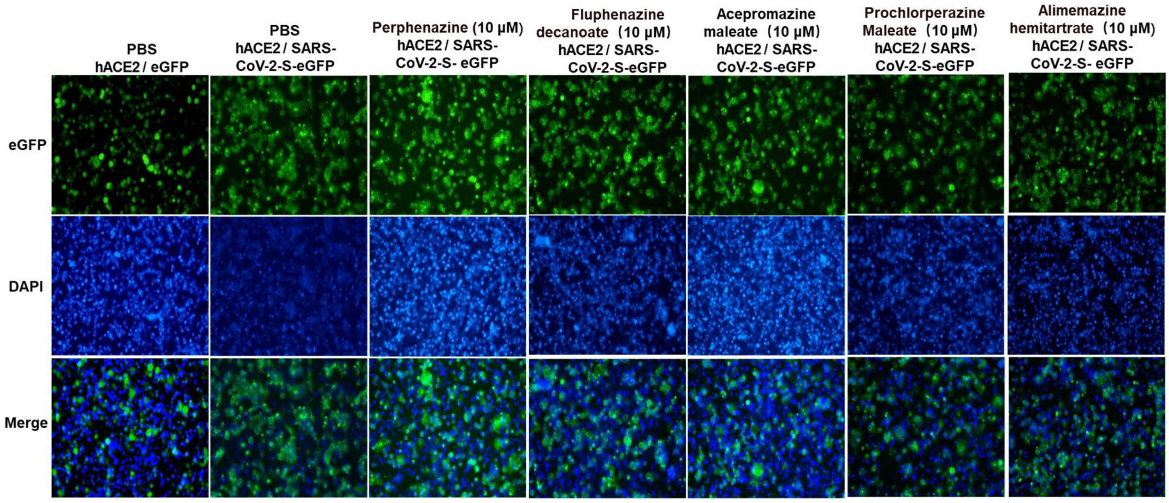

Figure S1. Images of SARS-CoV-2 S-protein-mediated cell–cell fusion in the presence of phenothiazines at the indicated concentration after co-culturing with 293T/SARS-CoV-2-eGFP and HEK293T-hACE2 cells for 12 h.

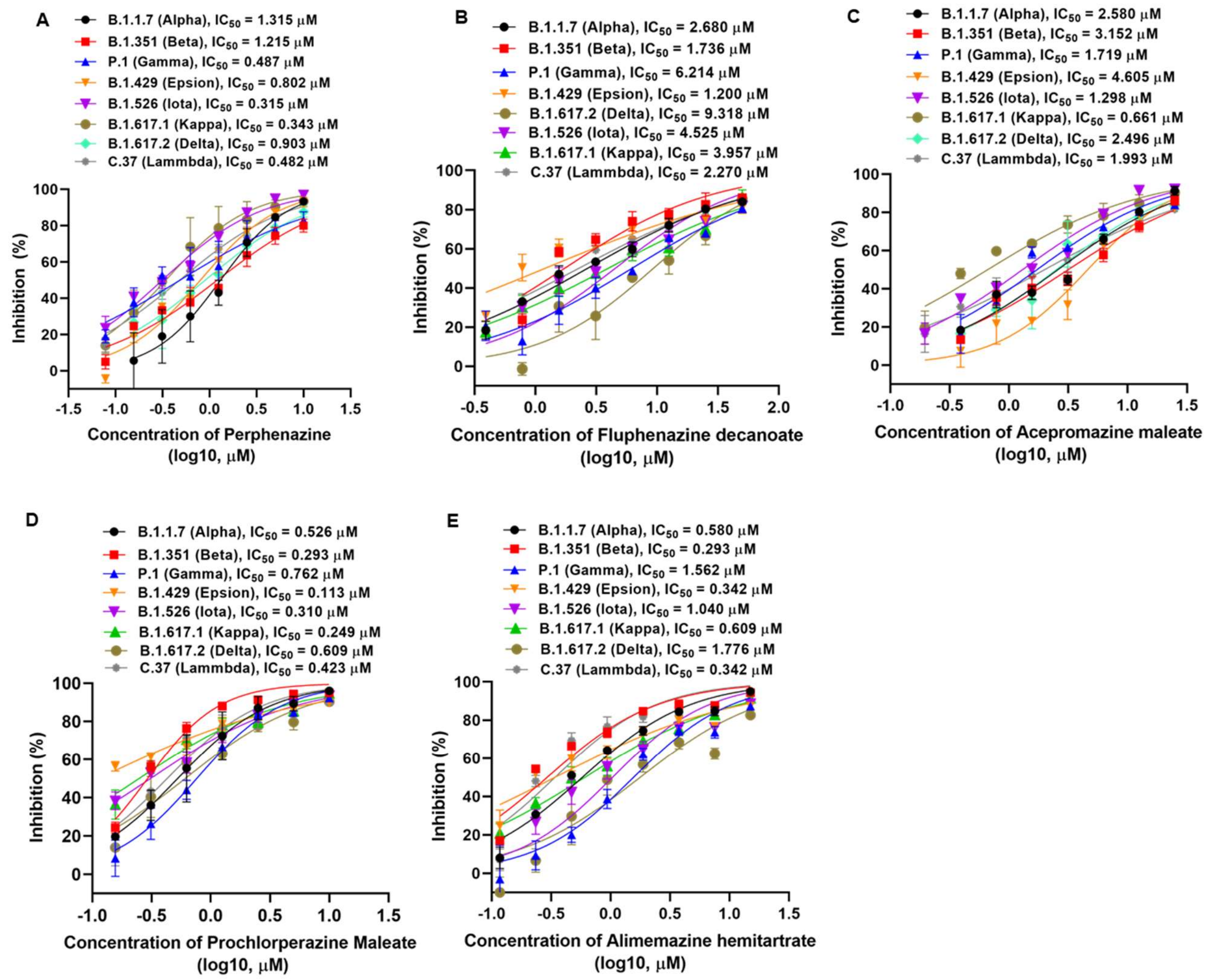

Figure S2. Inhibitory activities of phenothiazines against infection of pseudotyped SARS-CoV-2 subvariants. Efficacy of perphenazine (**A**), fluphenazine decanoate (**B**), acepromazine maleate (**C**), prochlorperazine maleate (**D**), and alimemazine hemitartrate (**E**) against B.1.1.7 (Alpha), B.1.351 (Beta), P.1 (Gamma), B.1.429 (Epsion), B.1.526 (lota), B.1.617.1 (Kappa), B.1.617.2 (Delta), and C.37 (Lambda) PsV infection. Samples were tested in triplicate, and the experiments were repeated at least twice. Data are presented in mean ± SD.
